# David Paul Brandes Goldberg, KBE, MD, FRCPsych (Hon)

**DOI:** 10.1192/bjb.2024.120

**Published:** 2025-08

**Authors:** Dinesh Bhugra

Formerly Professor of Psychiatry, Institute of Psychiatry, London, UK

**Figure:**
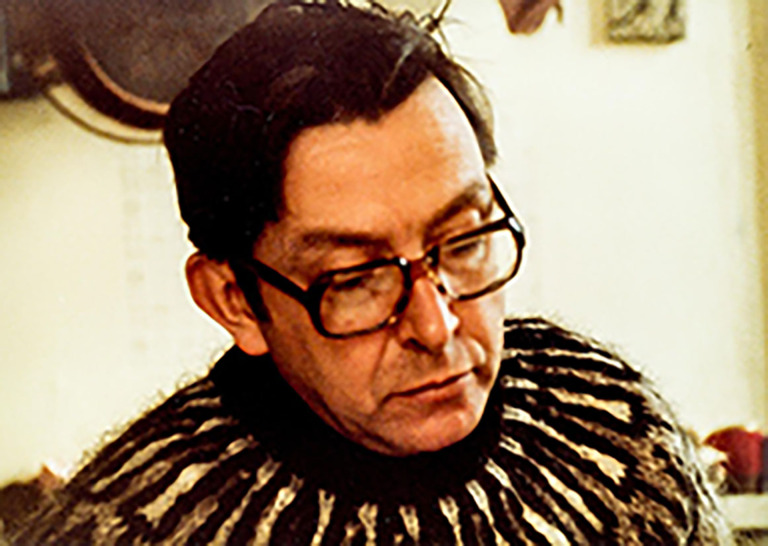

David Goldberg, who died on 5 September 2024, was a leading and highly influential figure in British psychiatry for over 50 years. The research achievement for which he is best known arose because of his interest in primary care psychiatry, to which he was introduced by Professor Michael Shepherd at the Institute of Psychiatry, London. There, in 1970, he developed the Clinical Interview Schedule as a structured interview, and in 1972, with Barry Blackwell, the General Health Questionnaire (GHQ) as a screening tool for the general population. This went through several versions. His research contributions to primary care psychiatry would continue for the rest of his life. Even after retirement he carried on with research in this area, advising the World Health Organization (WHO) on ICD for primary care and leading on surveys of mental health in primary care. As head of two major academic departments of psychiatry, both of which he expanded significantly, David had a major influence on research directions and on the careers of many leading psychiatrists and psychologists.

He was appointed to the Chair of Psychiatry at the University of Manchester in 1972. Under David's leadership, the Manchester department became a powerhouse of training and research in psychiatry, with a reputation as the ‘Maudsley of the North’. As Professor Bill Deakin, a colleague at Manchester, recalls:

‘The department was a vibrant and busy place with academic staff, secretaries and their offices, trainees, medical students and coffee room centred around the 100-seater lecture theatre. The Friday morning case conference was an occasion not to be missed; the lecture theatre full, the front row occupied by the academics and the distinguished consultant staff, David often chairing, a nervous trainee presenting the case, and then David interviewing the patient in his state-of-the-art TV studio relayed to the lecture theatre. David was very good at putting patients at their ease and conversationally clarifying symptoms and predicaments. The discussion afterwards was always lively and often inspirational, but it could evoke a certain frisson of anxiety, as at any time with David, due to his status and his erudition. However, most people figured out that fundamentally David was a gregarious, sociable person, and this contributed to the optimistic sense of purpose about the department.’

David felt that in psychiatry communication was the most important skill. He spent a year-long sabbatical at the University of South Carolina and brought back the practice of using video recordings of interviews by trainees as a teaching tool. He introduced communication skills training, during which trainees would produce videos of themselves interviewing patients and he would take them through the video virtually frame by frame. Working with medical psychotherapists, this approach became an incredibly popular and fruitful tool for teaching and learning. He worked with colleagues to develop psychiatric social work teaching, developing clinical psychology programmes and building a research base.

As Peter Huxley recalls:

‘He found the categorical approach to classification inappropriate for common mental disorders and took a dimensional approach which put him at odds with DSM et al. The overlap between symptoms of anxiety and depression lay at the heart of this view, and to my mind he has been proved right.’

Peter attributes David's influence on epidemiological studies and standardisation as crucial in the progress of psychiatry.

David returned to the Institute of Psychiatry (now called the Institute of Psychiatry, Psychology & Neuroscience), where he had trained in the 1960s, in 1993 and he remained there until retirement in 1999. I had the good fortune to be appointed to his department as a senior lecturer. He appointed Professors Peter Huxley, Kevin Gournay, André Tylee and many others across disciplines. He revitalised the Institute by introducing Maudsley Discussion Papers and reintroducing Maudsley Monographs, organising evening lectures open to the public and teaching trainees to be advocates for patients and psychiatry. He set up a department for health services research, housed in a building named after him. He was knighted in 1997.

During his tenure it was the 750th anniversary of the Bethlem Hospital (1997) and he commissioned Professor Roy Porter to write a book on the history of Bethlem; a celebratory conference was followed by dinner at the Natural History Museum. During this period, David chaired the Royal College of Psychiatrists’ Research Unit in an interim capacity. He had been Chair of the College's Joint Committee on Psychiatric Higher Training (JCHPT), which was responsible for carrying out visits on behalf of the College and approving training posts. When I instituted the RCPsych Awards in 2009, he was awarded the inaugural Lifetime Achievement award.

David was born in London on 28 January 1934. His father, Paul, was a civil servant and his mother, Ruby, a secretary. The family moved to Oxfordshire owing to the looming Second World War, leading to some disruption to his education. He was bullied in the new school as he was perceived to be an outsider. In his privately published memoirs, David recalled making exploding jam jars with calcium chloride and a little water to keep ‘our enemies in order and maybe treat us with greater respect’.

He remembered that while in the Upper Sixth in school, he decided he wanted to be a psychiatrist although it was not clear to him what a psychiatrist was. He went to the University of Oxford in 1952 and studied medicine. David's clinical placements were at St Thomas’ Hospital in London. He won the Exhibition in Psychological Medicine. In an interview with me for the book *Psychiatrists on Psychiatry: Conversations with Leaders*, he recalled the influence a paper on uncertainty in psychiatry by Sir Aubrey Lewis had on him and he moved to the Maudsley for training in psychiatry. During this period, he took exams for Membership of the Royal College of Physicians (London) but kept failing. He led the rebellion against the proposed new Royal College of Psychiatrists – following on from the Royal Medico-Psychological Association – because it was perceived to be interested in exams to make money like other royal colleges.

After retirement, he took over as the Chair of the Psychiatry Research Trust at the Institute of Psychiatry. He remained active in advising the WHO and running leadership training courses with Professor Norman Sartorius, who had been a friend for over 50 years. Norman recalls: ‘His knowledge and wit made it a pleasure to work with him and his devotion to work was infectious to teams which he joined’. When Norman started an educational programme focusing on leadership and other professional skills, he invited David to join in teaching young psychiatrists in European and other countries. Norman notes: ‘The students in these courses told me how helpful he was whenever approached for help. Other colleagues who worked with him or met him during our travels told me the same’.

Various colleagues noted that he could get impatient but ‘always productively’. He could be forceful, but his confidence and clarity of thought helped take people with him. As Peter Huxley recalls: ‘The words that spring to mind when I consider all of this are: Generosity; Loyalty (intense if he liked you); a naughty sense of humour and an inspiration’. He was always open to new ideas and debates. As Professor Gournay remarks: ‘On my arrival at the Institute, David gave me a very important piece of advice; saying: “Know what you don't know and never be afraid to ask”. I took him at his word and asked him many questions – he gave me much advice, always delivered very willingly and often accompanied with a humorous anecdote. To him I owe a great debt of gratitude’.

David met Ilfra Pink in St Thomas’ Hospital and they married in April 1966. She was medically qualified and, when they moved back to London from Manchester, she was appointed to London deanery to support the training of women doctors. They had four children and nine grandchildren. Ilfra died 7 years before he did. After her death, David lived with Dr Anne Geller, whom he had known as an undergraduate in Oxford. During the last 2 years of his life, he developed Alzheimer's disease, but retained his sense of mischief, fun and humour. I would visit him almost weekly at home and we would raise a glass of bubbly and drink to old times.

David is survived by his children, Paul, Charlotte, Kate and Emma, and grandchildren.

